# Peripheral eosinophil count and eosinophil-to-lymphocyte ratio are associated with revision sinus surgery

**DOI:** 10.1007/s00405-022-07497-2

**Published:** 2022-06-23

**Authors:** Karina Bayer, Selmir Hamidovic, Faris F. Brkic, Gerold Besser, Christian A. Mueller, David T. Liu

**Affiliations:** Department of Otorhinolaryngology, Head and Neck Surgery, Medical University of Vienna, Waehringer Guertel 18-20, 1090 Vienna, Austria

**Keywords:** Chronic rhinosinusitis, Endoscopic sinus surgery, CRS, ESS, Eosinophil-to-lymphocyte ratio, ELR, Eosinophils, ENR, NLR, BLR

## Abstract

**Purpose:**

The aim of this study was to assess whether hematological indices of the peripheral blood are associated with revision surgery in patients with chronic rhinosinusitis (CRS) undergoing endoscopic sinus surgery (ESS).

**Methods:**

This retrospective, cross-sectional, single-center study included 541 CRS patients that underwent ESS. Demographics and laboratory parameters were retrieved, and group comparisons were performed. We computed binary logistic regression models to associate clinical characteristics (i.e., aeroallergen allergies, asthma, presence of nasal polyps), absolute blood counts of different leukocyte types (i.e., lymphocytes, neutrophils, basophils, and eosinophils), and hematological indices of the peripheral blood [i.e., neutrophil-to-lymphocyte ratio (NLR), basophil-to-lymphocyte ratio (BLR), eosinophil-to-lymphocyte ratio (ELR), and eosinophil-to-neutrophil ratio (ENR)] with revision surgery as outcome.

**Results:**

The study population included 435 primary surgeries and 106 revision cases. Patients undergoing revision ESS showed significantly higher absolute preoperative eosinophil counts (381.6 ± 265.6 per µl), ELR (0.205 ± 0.195), and ENR (0.105 ± 0.074) compared to primary cases (all *p <* 0.001). Binary logistic regression analysis revealed higher odds for revision surgery in patients higher in age (*β* = 1.026, *p* < 0.001), with presence of aeroallergen allergies (*β* = 1.865, *p* = 0.011), presence of asthma (*β* = 3.731, *p* = 0.001), higher preoperative eosinophil counts (*β* = 1.002, *p* < 0.001), and higher ELR (*β* = 155.663, *p* = 0.015).

**Conclusions:**

We found that higher peripheral eosinophil count and higher ELR were associated with revision ESS in CRS patients. Preoperative assessment of hematological indices of the peripheral blood might be a valuable indicator of disease severity, thus facilitating better treatment selection.

## Introduction

Chronic rhinosinusitis (CRS) is a common global disease caused by inflammatory processes of the nose and the paranasal sinuses. The main treatment goal of CRS is the reduction of local inflammation, infection control, and the restoration of sinonasal mucociliary clearance. For patients that fail appropriate medical therapy, endoscopic sinus surgery (ESS) represents an appropriate treatment option [1]. However, up to 20% of patients undergoing primary ESS experience persistent post-treatment symptoms and require revision surgery [2–4]. The clinical subtype of CRS and other patient-specific factors affect the outcome and success of surgical treatment. Especially the factors of higher age, presence of nasal polyps (NP), physician-diagnosed asthma, and aeroallergen allergies were highly associated with revision surgery in previous studies [3, 5, 6]. Identifying prognostic parameters associated with revision surgery is essential to improve patients’ counseling in a way that calibrates expectations appropriately.

When pathogens enter the mucosal barrier, patients showcase different inflammatory endotypes, with type 1 being targeted at viruses, type 2 at parasites, and type 3 at fungi and bacteria [3]. Those inflammatory processes aim to restore the integrity of the mucosal barrier. In CRS patients, a pathogen is believed to cause a breach of the mucosal barrier, leading to a chronic inflammatory process, that fails to eliminate the causal pathogen. CRS patients exhibit one or more inflammatory patterns, with type 2 inflammation being the most common form [7]. Type 2 inflammation is characterized by increased production of interleukin 4 (IL-4), interleukin 5 (IL-5), interleukin 13 (IL-13), immunoglobulin E (IgE), and the recruitment and activation of mast cells and eosinophils [3, 8]. CRS patients that showcase type 2 inflammation as their predominant inflammation type were found to be more prone to therapy resistance and disease recurrence. Recent studies reported that type 2 inflammation causes imbalances between fibrinolytic and coagulative agents, which contributes to the formation of a fibrin mesh, which is suspected to be the primary driver in nasal polyp formation [9]. Modern biological agents can suppress the type 2 inflammatory patterns and could facilitate a personalized treatment for CRS patients based on their inflammatory type. Tissue eosinophilia and elevated blood eosinophil counts are markers for type 2 inflammation in CRS patients with and without nasal polyps and are associated with poor disease outcome [10, 11]. Such easily accessible markers are essential to facilitate personalized treatment of CRS patients.

Hematological indices of the peripheral blood, such as increased neutrophil-to-lymphocyte ratio (NLR), basophil-to-lymphocyte ratio (BLR), and eosinophil-to-lymphocyte ratio (ELR), were previously described as potential markers for disease recurrence in CRS patients [12, 13]. However, although elevated blood eosinophil levels were observed in CRS patients experiencing disease recurrence and treatment difficulties, it remains unclear whether the above-mentioned hematological indices of the peripheral blood and especially the ELR, which is derived from the blood eosinophil count, can be used as indicators for disease recurrence and revision ESS. Therefore, this study aimed to further elucidate the validity of those hematological indices of the peripheral blood to predict disease recurrence in CRS patients undergoing ESS with particular emphasis on peripheral eosinophil count and ELR.

## Materials and methods

### Data collection

This study followed a cross-sectional, single-center design and was approved by the Ethics committee of the Medical University of Vienna (EK-Nr.: 1736/2020). The study was conducted at the Department of Otorhinolaryngology at the Medical University of Vienna (MUV). We retrospectively included 541 patients diagnosed with CRS based on EPOS established criteria who underwent ESS at the MUV between 01.01.2012 and 31.12.2015 [3]. The patients’ demographics and clinical characteristics were obtained from the hospital’s patient record database. Demographics included age at the time of surgery (years) and gender (male/female). Clinical characteristics included smoking status (smoking/non-smoking), reported aeroallergen allergies (yes/no), physician-diagnosed diabetes (yes/no), and the presence of nasal polyps (yes/no) during the clinical examination.

### Laboratory parameters

All patients provided a routine blood test (i.e., complete blood count) preoperatively (0–45 days before surgery). On average, the peripheral blood samples were taken 8.8 days (SD = 6.2) prior to surgery. Blood samples were taken at a single point in two groups – prior to primary ESS in naïve cases and prior to revision ESS in recurrent cases. The absolute serum counts of lymphocytes, neutrophils, basophils, and eosinophils were obtained (absolute number/µl) and used to calculate the neutrophil-to-lymphocyte ratio (NLR), basophil-to-lymphocyte ratio (BLR), eosinophil-to-lymphocyte ratio (ELR), and eosinophil-to-neutrophil ratio (ENR).

### Statistical analysis

We performed statistical analyses using SPSS (IBM, Version 26.0 for MacOs, IBM Corp., Armonk, NY, USA). Continuous variables, such as eosinophil count (absolute number/µl) and ELR, were presented as mean ± standard deviation (SD). Categorical variables were presented as absolute numbers (percentages). We first divided our patients into two groups to further elucidate differences between patients who had not undergone ESS (primary surgery) and patients who had already received surgical treatment of the paranasal sinuses (revisions). We used two-tailed unpaired *t*-tests to facilitate mean comparisons for the hematological indices of the peripheral blood and other normally distributed continuous variables. We applied Chi-squared tests (*χ*^2^-test) for group comparisons of binary categorical variables.

We performed univariate binary logistic regression analysis with revision surgery as the outcome and the factors of age (in years), gender (reference: female), smoking (reference: non-smoker), aeroallergen allergies (reference: not present), asthma (reference: not present), presence of nasal polyps (reference: not present), lymphocyte count (absolute number/µl), neutrophil count (absolute number/µl), basophil count (absolute number/µl), and eosinophil count (absolute number/µl) as explanatory variables. Two multivariable binary logistic regression models with revision surgery as outcome were computed: the first model included the different counts (absolute number/µl) of leukocytes (i.e., lymphocyte count, neutrophil count, basophil count, and eosinophil count) and clinical characteristics (i.e., age, gender, smoking, aeroallergen allergies, asthma, presence of nasal polyps) as predictive variables, while the second model included the hematological indices of the peripheral blood (i.e., NLR, BLR, ELR, and ENR) and clinical characteristics (i.e., age, gender, smoking, aeroallergen allergies, asthma, presence of nasal polyps) as predictive variables. The adjusted odds ratios (*β*) and 95% confidence intervals (CI) were calculated for each variable. The level of significance was set at 0.05.

## Results

### Patient selection

Five hundred and forty-one patients who underwent ESS at the MUV were eligible for this study throughout the observational period. This study population was partly included in an earlier study [6]. Four hundred and thirty-five patients (80.4%) underwent primary surgery (i.e., no previous sinus surgery had been performed) and 106 patients (19.6%) underwent revision ESS (i.e., the patient had previously undergone one or more sinus surgeries). The study population comprised 244 (45.1%) women and 297 (54.9%) men with a mean age of 42.0 years (SD = 15.4 years). Of all included cases, 171 (31.6%) patients reported aeroallergen allergies, and 35 (6.5%) had been diagnosed with asthma. Nasal polyps were present in 389 (71.9%) cases (Table 1). Unpaired two-tailed *t*-test revealed that patients undergoing revision surgery were significantly older than patients undergoing primary surgery (*p*-value: 0.001). *χ*^2^-test further revealed that patients undergoing revision surgery more frequently reported aeroallergen allergies (*p*-value < 0.001), physician-diagnosed asthma (*p*-value < 0.001), and the presence nasal polyps (*p*-value = 0.034).

**Table 1:** Study participants’ demographical characteristics

Demographics	All cases (*N* = 541)	Primary surgeries (*N* = 435)	Revisions (*N* = 106)	*p* value*
Age (in years)	42.0 ± 15.4	40.9 ± 15.6	46.5 ± 13.8	**0.001**^**a**^
Gender, female	244 (45.1%)	204 (46.9%)	40 (37.7%)	0.089^**b**^
Smoking, yes	155 (28.7%)	127 (29.2%)	28 (26.4%)	0.570^**b**^
Allergy, yes	171 (31.6%)	121 (27.8%)	50 (47.2%)	** < 0.001**^**b**^
Asthma, yes	35 (6.5%)	16 (3.7%)	19 (17.9%)	** < 0.001**^**b**^
Nasal polyps, yes	389 (71.9%)	304 (69.9%)	85 (80.2%)	**0.034**^**b**^

Continuous variables are presented as mean ± SD (standard deviation), categorical variables as absolute number (percentage)

*p value denotes group differences between primary and revision surgeries, bold values of p are significant

^a^Two-tailed *t*-test

^b^*χ*^2^-test

### Differences in laboratory parameters between primary and revision cases

In a first step, we were interested to know whether there were differences in preoperative peripheral blood counts of lymphocytes, neutrophils, basophils, eosinophils, NLR, BLR, ELR, and ENR between patients undergoing primary surgery and those undergoing revision ESS. We, therefore, performed unpaired t-tests between the primary and revision group.

**Fig. 1:**
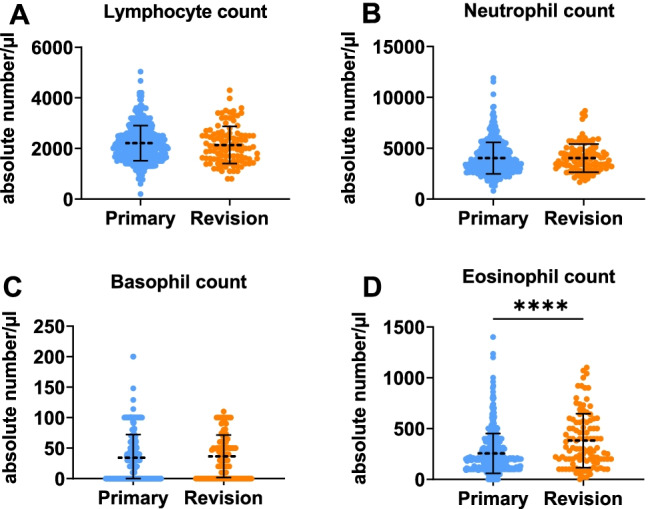
Scattergrams (mean ± SD) of the absolute serum counts of different leukocyte types. The primary surgery group (Primary) consisted of 435 CRS patients and the revision ESS group (Revision) of 106 patients. Groups were compared by unpaired *t*-tests. **A** lymphocyte count, **B** neutrophil count, **C** basophil count, **D** eosinophil count. ****p < 0.05

**Fig. 2:**
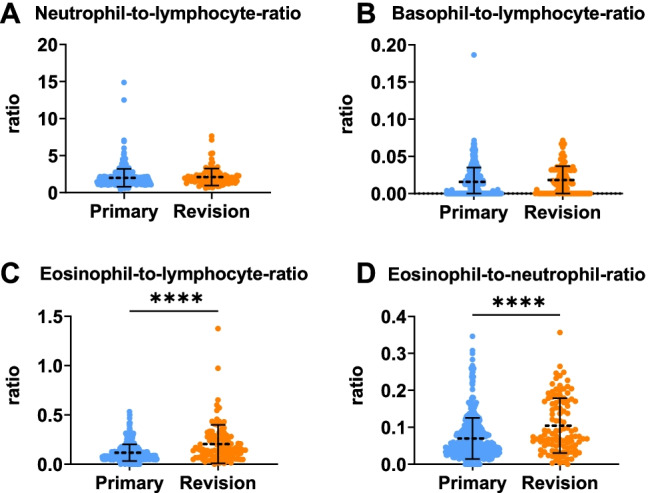
Scattergrams (mean ± SD) of the hematological indices of the peripheral blood. The primary surgery group (Primary) consisted of 435 CRS patients and the revision ESS group (Revision) of 106 patients. Groups were compared by unpaired *t*-test. **A** neutrophil-to-lymphocyte ratio (NLR), **B** basophil-to-lymphocyte ratio, **C** eosinophil-to-lymphocyte ratio (ELR), and **D** eosinophil-to-neutrophil ratio (ENR). ****p < 0.05

Unpaired *t*-tests revealed that the eosinophil-count was significantly higher in patients requiring revision ESS (*p*-value < 0.001) (Fig. 1). The ELR was significantly higher in patients undergoing revision surgery compared to primary surgeries (*p*-value < 0.001). Furthermore, the ENR was also significantly higher in patients undergoing revision surgery (*p*-value < 0.001). We found no significant differences in the NLR and the BLR between patients undergoing primary and revision ESS (Table 2, Fig. 2).

**Table 2:** All laboratory parameters listed as mean ± SD

Laboratory parameters	All cases (*N* = 541)	Primary surgeries (*N* = 435)	Revisions (*N* = 106)	*p* value*
Lymphocytes	2197.7 ± 702.0	2211.7 ± 694.5	2140.0 ± 732.2	0.346^a^
Neutrophils	4032.2 ± 1517.6	4033.9 ± 1550.0	4025.3 ± 1383.4	0.958^a^
Basophils	35.8 ± 44.6	35.5 ± 46.7	36.6 ± 34.9	0.822^a^
Eosinophils	279.9 ± 216.8	255.2 ± 195.6	381.6 ± 265.6	** < 0.001**^**a**^
Neutrophil-to-lymphocyte ratio	2.021 ± 1.196	1.999 ± 1.208	2.114 ± 1.149	0.376^a^
Basophil-to-lymphocyte ratio	0.016 ± 0.019	0.016 ± 0.019	0.018 ± 0.018	0.215^a^
Eosinophil-to-lymphocyte ratio	0.134 ± 0.120	0.117 ± 0.084	0.205 ± 0.195	** < 0.001**^**a**^
Eosinophil-to-neutrophil ratio	0.077 ± 0.061	0.070 ± 0.056	0.105 ± 0.074	** < 0.001**^**a**^

Lymphocytes, neutrophils, basophils, and eosinophils are listed as absolute number/µl

*p value denotes group differences between primary and revision surgeries, bold values of p are significant

### Factors associated with revision sinus surgery

As we found significant differences in peripheral eosinophil counts, ELR, and ENR between patients undergoing primary and revision surgery, we were next interested in knowing which factors were associated with revision surgery. For further assessment of the association of the above-mentioned peripheral blood markers with revision surgery, univariate and multivariable binary logistic regression analysis models were computed.

Univariate analysis revealed the factors of higher age (*β* = 1.02, 95% CI 1.01–1.04), presence of aeroallergen allergies (*β* = 2.32, 95% CI 1.50–3.58), asthma (*β* = 5.72, 95% CI 2.83–11.56), and nasal polyps (*β* = 1.74, 95% CI 1.04–2.93) to be significantly associated with higher odds for revision surgery. Furthermore, higher peripheral blood eosinophil counts (*β* = 1.00, 95% CI 1.00–1.00), higher ELR (*β* = 335.01, 95% CI 47.16–2379.77), and higher ENR (*β* = 3182.88, 95% CI 132.02–76,736.86) were also significantly associated with revision ESS (Tables 3 and 4).

**Table 3:** Results of univariate and multivariable binary logistic regression analysis with the factors of age (in years), gender (reference: female), smoking (reference: non-smoker), allergies (reference: not present), asthma (reference: not present), nasal polyps (reference: not present), lymphocytes (absolute number/µl), neutrophils (absolute number/µl), basophils (absolute number/µl), and eosinophils (absolute number/µl) as explanatory variables to generate statistical estimates to calculate adjusted odds ratios (β) and 95% confidence intervals (CI)

	Univariate analysis		Multivariable analysis	
	*β* (95% CI)	*p* value*	*β* (95% CI)	*p* value*
Age (years)	1.023 (1.009–1.037)	**0.001**	1.027 (1.011–1.043)	**0.001**
Gender	1.457 (0.943–2.252)	0.090	1.581 (0.982–2.546)	0.059
Smoking	0.871 (0.539–1.405)	0.570	1.454 (0.839–2.517)	0.182
Allergy	2.317 (1.499–3.580)	** < 0.001**	1.913 (1.159–3.159)	**0.011**
Asthma	5.719 (2.829–11.563)	** < 0.001**	3.961 (1.771–8.861)	**0.001**
Nasal polyps	1.744 (1.037–2.933)	**0.036**	1.323 (0.756–2.314)	0.327
Lymphocytes (absolute number/µl)	1.000 (1.000–1.000)	0.345	1.000 (0.999–1.000)	0.076
Neutrophils (absolute number/µl)	1.000 (1.000–1.000)	0.958	1.000 (1.000–1.000)	0.449
Basophils (absolute number/µl)	1.001 (0.996–1.005)	0.822	0.996 (0.990–1.003)	0.265
Eosinophils (absolute number/µl)	1.002 (1.001–1.003)	** < 0.001**	1.002 (1.001–1.003)	** < 0.001**

*Bold values of *p* are significant

**Table 4:** Results of univariate and multivariable binary logistic regression analysis with the factors of age (years), gender (reference: female), smoking (reference: non-smoker), allergies (reference: not present), asthma (reference: not present), nasal polyps (reference: not present), neutrophil-to-lymphocyte ratio (NLR), basophil-to-lymphocyte ratio (BLR), eosinophil-to-lymphocyte ratio (ELR), and eosinophil-to-neutrophil ratio (ENR) as explanatory variables to generate statistical estimates to calculate adjusted odds ratios (β) and 95% confidence intervals (CI)

	Univariate analysis		Multivariable analysis	
	*β* (95% CI)	*p* value^+^	*β* (95% CI)	*p* value^+^
Age (years)	1.023 (1.009–1.037)	**0.001**	1.026 (1.010–1.042)	**0.001**
Gender	1.457 (0.943–2.252)	0.090	1.550 (0.960–2.504)	0.073
Smoking	0.871 (0.539–1.405)	0.570	1.379 (0.813–2.340)	0.233
Allergies	2.317 (1.499–3.580)	** < 0.001**	1.865 (1.128–3.083)	**0.015**
Asthma	5.719 (2.829–11.563)	** < 0.001**	3.731 (1.677–8.305)	**0.001**
Nasal polyps	1.744 (1.037–2.933)	**0.036**	1.246 (0.709–2188)	0.445
Neutrophil-to-lymphocyte-ratio	1.075 (0.915–1.263)	0.380	0.990 (0.767–1.279)	0.940
Basophil-to-lymphocyte-ratio	734.983 (0.020–26,872,168.8)	0.218	0.009 (0.000–4625.810)	0.483
Eosinophil-to-lymphocyte-ratio	335.010 (47.161–2379.768)	** < 0.001**	155.663 (2.663–8636.862)	**0.015**
Eosinophil-to-neutrophil-ratio	3182.876 (132.019–76,736.860)	** < 0.001**	1.217 (0.001–1586.037)	0.957

^+^Bold values of *p* are significant

As we found significant associations in univariate analysis, we next wanted to know which variables remained associated in multivariable analysis after controlling for demographics and clinical characteristics. In the multivariable model including age, gender, smoking status, presence of aeroallergen allergies, presence of physician-diagnosed asthma, presence of nasal polyps, absolute count of lymphocytes, neutrophils, basophils, and eosinophils we found that higher age (*β* = 1.03, 95% CI 1.01–1.04), aeroallergen allergies (*β* = 1.91, 95% CI 1.16–3.16), physician-diagnosed asthma (*β* = 3.96, 95% CI 1.77–8.86), and higher eosinophil count (*β* = 1.00, 95% CI1.00–1.00) were associated with revision surgery. The other model including age, gender, smoking status, presence of aeroallergen allergies, presence of physician-diagnosed asthma, presence of nasal polyps, and the hematological indices of the peripheral blood (i.e., NLR, BLR, ELR, and ENR) revealed that higher age (β = 1.03, 95% CI 1.01–1.04), aeroallergen allergies (*β* = 1.87, 95% CI 1.13–3.08), physician-diagnosed asthma (*β* = 3.73, 95% CI 1.68–3.31), and higher ELR (*β* = 155.66, 95% CI 2.66–8636.86) were associated with higher odds for revision ESS.

## Discussion

CRS is an inflammatory disease of the nose and paranasal sinuses and affects approximately 10.9% of the European population, creating a significant economic burden for healthcare with adverse effects on patients’ quality of life (QOL) [1, 2, 14]. CRS patients require ESS when adequate medical treatment is unsuccessful. However, up to 20% of patients undergoing ESS experience disease recurrence and require one or several revision surgeries, which is frequently associated with complications, lengthy treatment, and compromised QOL [3]. Therefore, it is of interest to elucidate factors associated with revision ESS and disease recurrence. Identifying peripheral blood markers, which can be used as predictive markers for CRS patients, is a simple, minimally invasive, and inexpensive way to facilitate customized treatment.. Elevated blood eosinophil counts were previously discussed as markers for type 2 inflammation in CRS patients with and without nasal polyps and are associated with poor disease outcome [11]. However, whether hematological indices of the peripheral blood (i.e., NLR, BLR, ELR, and ENR) are associated with disease recurrence and revision ESS remains unclear. This study found that patients undergoing revision ESS presented significantly higher absolute eosinophil counts and ELR in peripheral blood samples. Univariate binary logistic regression analysis revealed that the factors of higher age (years), presence of aeroallergen allergies, physician-diagnosed asthma, presence of nasal polyps, higher absolute eosinophil count (absolute number/µl), higher ELR, and higher ENR were significantly associated with revision surgery. Multivariable binary logistic regression analysis confirmed higher age, presence of aeroallergen allergies, physician-diagnosed asthma, higher absolute eosinophil count (absolute number/µl), and higher ELR as factors associated with higher odds for revision ESS.

The present study revealed that patients undergoing revision ESS showcased significantly elevated peripheral eosinophil blood counts and ELR compared to patients undergoing primary surgery. These findings align with previous studies, which reported that tissue eosinophilia was associated with increased disease severity and recurrence in CRS patients [15, 16]. The ELR was previously found to be significantly higher in CRS patients with nasal polyps experiencing disease recurrence and severe nasal polyposis [13]. This phenomenon has previously been explained by type 2 inflammatory patterns, which are associated with tissue eosinophilia and elevated blood counts of eosinophils. This suggests that peripheral eosinophil counts and ELR could serve as a predictor for severe cases of CRS and disease recurrence, which is supported by the results of this study.

Our findings did not reveal significant differences between primary surgeries and revisions for the other hematological indices of the peripheral blood (i.e., NLR and BLR). However, a previous study reported higher NLR and BLR in CRS patients with nasal polyps and disease recurrence, as elevated neutrophils and lymphocytes are indicators of inflammatory processes and basophils have been associated with inflammatory exacerbations [13]. A possible explanation for this finding is that our study did not exclusively include CRS patients with nasal polyps, unlike the study mentioned above.

Regarding demographics, higher age was another factor that had a significant association with revision surgery, which is in accordance with previous studies that reported higher age as a risk factor for revision ESS in CRS patients [3]. However, gender and smoking status did not show significant differences between primary and revision cases, although previous studies reported that female gender is associated with revision surgery [3]. Similarly, smoking was found to shorten the time between primary and revision surgeries in CRS patients with nasal polyps and to be more prevalent among patients undergoing revision ESS [17, 18]. This phenomenon might be caused by the deleterious effects of smoke on the sinonasal mucosa, which contributes to polyp regrowth. The discrepancy in smoking status might be explained by the fact that this study only distinguished between smokers and non-smokers and did not consider previous smoking. Concerning gender, this study included fewer women (45.1%) than men, which, in combination with the cross-sectional design of this study, could have contributed to the discrepancy concerning gender.

Concerning the impact of aeroallergen allergy, we found that patients undergoing revision ESS reported aeroallergen allergies significantly more frequently than patients undergoing primary surgery. This finding was not surprising, as aeroallergen allergies have previously been associated with disease recurrence in CRS patients [3]. Furthermore, we found that patients diagnosed with asthma required revision ESS significantly more frequently than patients without asthma. This is in accordance with earlier studies that also reported higher revision rates in  CRS patients with asthma [3, 5, 19]. Previously asthma was found to be related to disease recurrence in CRS patients with and without nasal polyps alike, which emphasizes the importance of asthma as a prognostic tool in CRS patients [19]. Aeroallergen allergies and asthma are both associated with type 2 inflammatory patterns, which contribute to nasal polyp formation [9]. This is also in accordance with the united airways hypothesis, which states that a single inflammation can cause diseases of the upper and lower airways, as the inflammatory process distributes across the upper and lower airways, which are considered a single organ [20]. Aeroallergen allergies and asthma thus represent an important clinical marker for CRS patients.

Although group comparisons and univariate binary logistic regression analysis revealed a significant association between the presence of nasal polyps and disease recurrence, this factor failed to reach significance in our multivariable binary logistic regression model. Nevertheless, the EPOS guidelines and previous studies list nasal polyposis as an essential risk factor for disease recurrence and the demand for revision ESS [3, 8]. This partial discrepancy to the literature might be explained by the fact that nasal polyps are associated with type 2 inflammation, which is linked to elevated eosinophil counts. Therefore, elevated eosinophil levels and type 2 inflammation might be more strongly  associated with revision surgery than the presence of nasal polyps alone.

This study provides further evidence for the prognostic validity of the preoperative evaluation of hematological indices of the peripheral blood, such as the ELR for CRS patients and allowed for an assessment of potential risk factors for a relatively large cohort of CRS patients undergoing primary and revision ESS. However, the limitations of this study include the single-center and cross-sectional design, which did not allow the inclusion of patients undergoing revision ESS at other hospitals throughout the observational period and beyond. As this study followed a cross-sectional design, we only observed patients at a single point (i.e., before surgery) and did not gain insight into, whether the patients required revision surgery or experienced disease recurrence after the study period. Future studies could follow a longitudinal design and would contribute to our current level of knowledge by further investigating the individual patient’s course of disease and revision rates. However, using revision surgery as the outcome implies a certain bias in itself, as some patients experience disease recurrence, but abstain from undergoing revision surgery or undergo surgery with significant time delay because of risk or fear of complications, inability to undergo anesthesia, due to medical conditions, or other reasons. Nevertheless, we obtained further insight into the validity of preoperative hematological indices of the peripheral blood and identified the ELR as a possible future predictor of disease severity and recurrence in CRS patients.

## Conclusions

We found that peripheral eosinophil count and ELR were significantly higher in patients experiencing disease recurrence, suggesting that these parameters could serve as indicators for severe cases of CRS. Our results suggest that the routine evaluation of peripheral eosinophil counts and the calculation of the ELR could be used to adapt treatment to the individual CRS patient’s demand and facilitate a better selection of treatment options, including biological agents targeted to suppress type 2 inflammatory patterns.
